# Protein tyrosine kinase Abl promotes hepatitis C virus particle assembly *via* interaction with viral substrate activator NS5A

**DOI:** 10.1016/j.jbc.2022.101804

**Published:** 2022-03-04

**Authors:** Daisuke Miyamoto, Kenji Takeuchi, Kazuyasu Chihara, Shigeharu Fujieda, Kiyonao Sada

**Affiliations:** 1 Department of Otorhinolaryngology Head & Neck Surgery, Faculty of Medical Sciences, University of Fukui, Fukui, Japan; 2 Department of Genome Science and Microbiology, Faculty of Medical Sciences, University of Fukui, Fukui, Japan; 3 Organization for Life Science Advancement Programs, University of Fukui, Fukui, Japan

**Keywords:** protein tyrosine kinase, phosphotyrosine, host factors against virus infection, assembly of infectious virus particle, Abl tyrosine kinase, hepatitis C virus, viral protein, nonstructural protein 5A, Abl, Abelson, Abl^−^, Abl-deficient cell, AFK, Abl family kinase, cDNA, complementary DNA, DAA, direct-acting antiviral, DOC, sodium deoxycholate, DSL, detergent-soluble lysate, edR, editing-resistant, ER, endoplasmic reticulum, HCV, hepatitis C virus, HCVcc, cell culture–adapted HCV, HEK 293T, human embryonic kidney 293T cell line, IgG1, immunoglobulin G1, KD, kinase-dead, mAb, monoclonal antibody, NS5A, nonstructural protein 5A, pTyr, phosphotyrosine, SH3, Src homology 3, TCID_50_, median tissue culture infectious dose

## Abstract

Previously, we reported that knockdown of Abl protein tyrosine kinase by shRNA or pharmacological inhibition suppresses particle assembly of J6/JFH1 strain–derived hepatitis C virus (HCV) in Huh-7.5 cells. However, the detailed mechanism by which Abl regulates HCV replication remained unclear. In this study, we established Abl-deficient (Abl^−^) cells through genome editing and compared HCV production between Abl^−^ cells expressing WT or kinase-dead Abl and parental Huh-7.5 cells. Our findings revealed that Abl expression was not required from the stages of virus attachment and entry to viral gene expression; however, the kinase activity of Abl was necessary for the assembly of HCV particles. Reconstitution experiments using human embryonic kidney 293T cells revealed that phosphorylation of Tyr^412^ in the activation loop of Abl was enhanced by coexpression with the viral nonstructural protein 5A (NS5A) and was abrogated by the substitution of NS5A Tyr^330^ with Phe (Y330F), suggesting that NS5A functions as a substrate activator of Abl. Abl–NS5A association was also attenuated by the Y330F mutation of NS5A or the kinase-dead Abl, and Abl Tyr^412^ phosphorylation was not enhanced by NS5A bearing a mutation disabling homodimerization, although the association of Abl with NS5A was still observed. Taken together, these results demonstrate that Abl forms a phosphorylation-dependent complex with dimeric NS5A necessary for viral particle assembly, but that Abl is capable of complex formation with monomeric NS5A regardless of tyrosine phosphorylation. Our findings provide the foundation of a molecular basis for a new hepatitis C treatment strategy using Abl inhibitors.

Hepatitis C virus (HCV), an enveloped positive-strand RNA virus and a member of the Flaviviridae family (1, 2, 3), is a frequent cause of acute/chronic hepatitis, liver cirrhosis, and hepatocellular carcinoma in humans. HCV binds to the target cell membrane and enters the cell *via* clathrin-mediated endocytosis (4, 5), where the viral particle releases genomic RNA, which is translated to a polyprotein of 3010 to 3014 amino acids in the endoplasmic reticulum (ER) because the viral RNA also functions as an mRNA for the viral polyprotein (2). This polyprotein is post-translationally cleaved by both viral and host proteases to form three structural proteins (core, E1, and E2) and seven nonstructural (NS) proteins (p7, NS2, NS3, NS4A, NS4B, NS5A, and NS5B). The NSs form the RNA replication complex on ER-derived membranous compartments (6). The RNA replication complex synthesizes positive-strand RNA, which is assembled with structural proteins into a virus particle through budding into the ER lumen (1, 3). The progeny viral particles pass through the Golgi apparatus, reach the cell membrane, and are released from the infected cells.

NS5A is believed to be involved in HCV RNA replication and infectious virus particle formation and assembly (3). NS5A is a phosphoprotein that exists in low-phosphorylated and high-phosphorylated forms (7, 8) and contains domain I, which includes an N-terminal amphiphilic α-helix, domain II, and domain III separated by two low-complexity sequences (9) (Fig. 1*A*). Domain I and parts of domain II are involved in viral genome replication (9, 10, 11, 12), whereas most of domain II and domain III are involved in viral particle assembly (13, 14). Domain I contains four cysteine residues (Cys^39^, Cys^57^, Cys^59^, and Cys^80^), which mediate zinc binding at the N terminus (9, 15). The ability of the protein to homodimerize is lost following substitution of any one of the cysteine residues with Ala (12). Domain I was reported to be involved in the production of infectious viral particles (16, 17). Phosphorylation of Tyr^93^ in domain I is important for formation of the viral genome replication complex, and Src, a nonreceptor tyrosine kinase, is involved in this phosphorylation (18).

**Figure 1:**
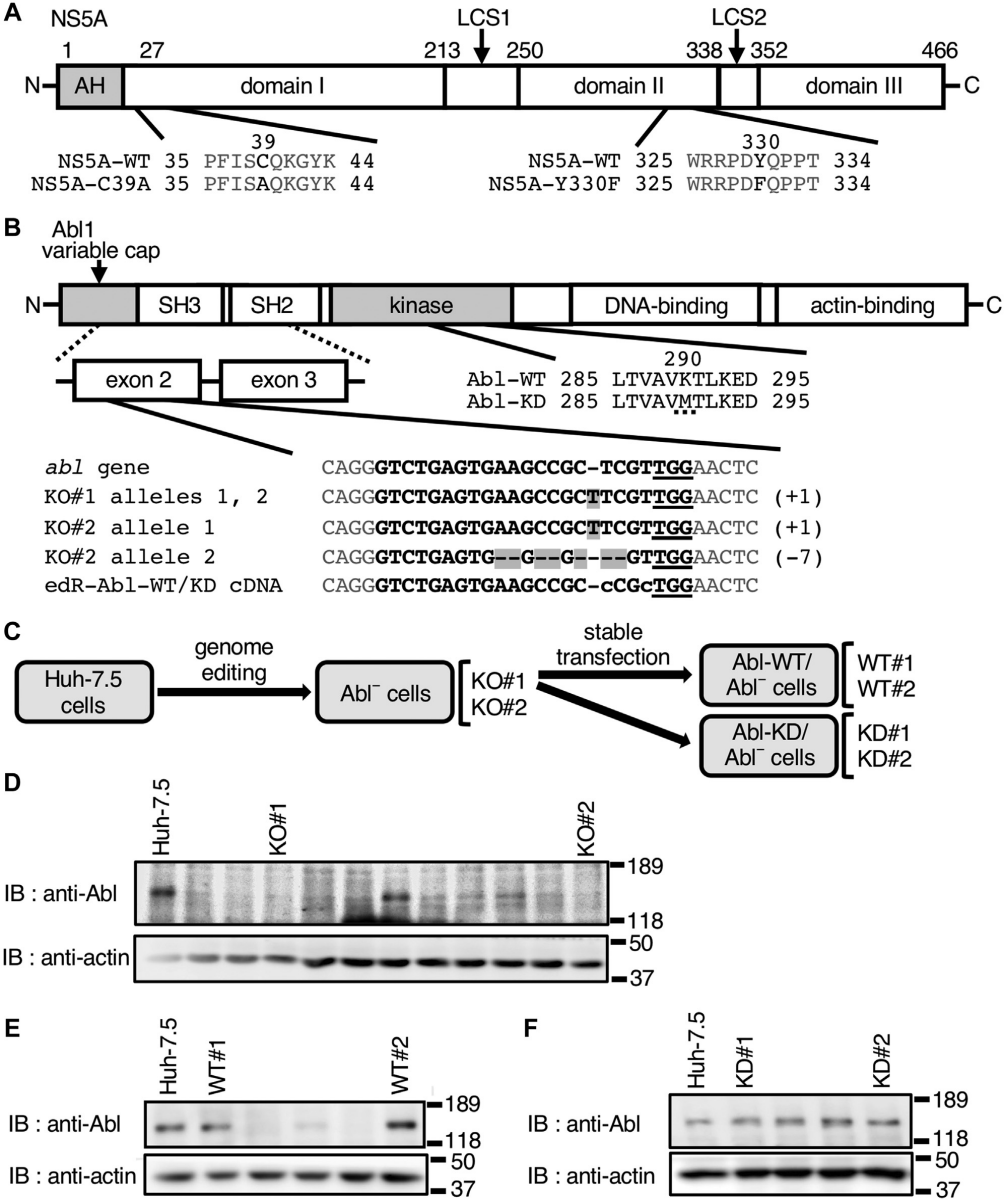
**Mutant proteins and cells used in this study.***A*, structure of NS5A and amino acid substitutions in the NS5A mutants used in this study. NS5A has an amphiphilic N-terminal α-helix (AH) and domains I, II, and III separated by two low-complexity sequences (LCS I and II). Amino acid sequences of NS5A mutants (C39A and Y330F) are shown. Numbers in the figure indicate the amino acids of NS5A. *B*, structures of Abl and its variants used in this study. Abl has a variable cap structure and SH3, SH2, and tyrosine kinase domains at the N-terminal region and the DNA-binding and actin-binding domains at the C-terminal region. The substitution of Lys^290^ with Met (*dotted underline*) in Abl-KD is shown in the *middle*. Comparison of the nucleotide sequences of *abl* in Huh-7.5 cells, Abl^−^ cells (KO#1 and KO#2), and edR-Abl-WT/KD complementary DNA is shown at the *bottom of the panel*. One mutation type was identified in KO#1 cells, and two mutation types (allele 1 and allele 2) were identified in KO#2 cells. The *shaded letters* are mutations caused by genome editing. The regions flanked by *gray letters* are the target sequence of guide RNA. *Underlined letters* indicate protospacer adjacent motif (PAM) sequences. *C*–*F*, establishment of Abl mutant cells. *C*, we established two independent Abl^−^ cell lines (KO#1 and KO#2) from parental Huh-7.5 cells through genome editing. Abl-add-back cells (Abl-WT/Abl^−^ and Abl-KD/Abl^−^ cells) were generated *via* the stable transfection of edR forms of complementary DNA into KO#1 cells. Two WT- and KD-expressing cell lines each were selected for further analysis (WT#1 and WT#2 or KD#1 and KD#2). Generation of Abl^−^ (*D*), Abl-WT/Abl^−^ (*E*), and Abl-KD/Abl^−^ (*F*) cells. The DSL of Huh-7.5 cells and cloned cells was separated *via* SDS-PAGE and analyzed *via* immunoblotting with the anti-Abl mAb, with antiactin mAb as an internal control. Molecular size markers are shown on the *right*. Abl, Abelson; Abl^−^, Abl deficient; DSL, detergent-soluble lysate; edR, editing-resistant; KD, kinase-dead; mAb, monoclonal antibody; NS5A, nonstructural protein 5A.

The Abl family kinases (AFKs) are a family of nonreceptor tyrosine kinases comprising Abl (c-Abl or Abl1) and Arg (Abl2) of vertebrate origin (19). Abl was discovered as an intracellular homolog of v-Abl, a tumor protein of Abelson murine lymphosarcoma virus (20). Abl is a ubiquitously expressed signaling molecule that regulates cell proliferation, differentiation, adhesion, morphogenesis, polarity, migration, and invasion and is also involved in cancer (19). Most patients with chronic myeloid leukemia and some with acute myeloid leukemia have a chromosomal abnormality called the Philadelphia chromosome. This somatic translocation, t(9:22), gives rise to a deregulated fusion tyrosine kinase called BCR–Abl (p210^Bcr–Abl^), which is the molecular target of leukemia therapy with imatinib (21). The N terminus of Abl contains a cap region (in the case of variant b of Abl, a myristoyl chain), which maintains the kinase in a locked and inactive conformation; the Src homology 3 (SH3) domain, SH2 domain, and catalytic tyrosine kinase domain (SH1). The C terminus contains the DNA-binding and actin-binding domains (Fig. 1*B*). The SH3 domain binds to the polyproline-containing linker sequence that connects the SH2 domain to the kinase domain, and the SH2 domain associates with the C-lobe of the kinase domain to form the inactive “clamped” conformation (22). The presence of the myristate-binding protein, SH3-binding protein, SH2-binding protein, and Tyr^412^ phosphorylation in the activation loop of the kinase domain induces a change into the active “unclamped” conformation of Abl (21, 23, 24).

We have previously reported that knocking down Abl expression using shRNA or treatment with an Abl inhibitor (imatinib) suppresses HCV replication at the stage of viral particle assembly and that Abl is involved in the tyrosine phosphorylation of NS5A (25). Pox virus (26) and Ebola virus (27) utilize Abl for virus production, and the possibility of using Abl inhibitors against these viruses has been previously discussed (26, 27, 28). However, in these viruses, the formation of viral particles occurs on the plasma membrane. By contrast, HCV particle assembly occurs on the ER membrane, and the assembled virus particles are secreted to the extracellular space *via* the Golgi apparatus (1, 3). Thus, it remains unclear whether HCV and the other two viruses utilize Abl through the same mechanism.

To clarify the involvement of Abl in HCV particle assembly, we established various mutant cell lines from Huh-7.5 cells. This experimental system (Fig. 1*C*) is different form that used in the previous study (25). In addition, we investigated the interaction of Abl with NS5A as a target molecule and the mechanism of their interaction using a reconstituted human embryonic kidney 293T (HEK 293T) cell system.

## Results

### Generation and expression analysis of various Abl-expressing mutant cell lines

G418-resistant clones were screened for Abl expression through immunoblotting, and several clones were obtained (Fig. 1*D*). Two clones (KO#1 and KO#2) were selected for sequencing of genomic PCR fragments covering the exon 2 of *abl*. A frameshift mutation was detected in the genomes of KO#1 and KO#2 cells, although the base mutations were the same (Fig. 1*B*). Blasticidin-resistant clones were screened for Abl expression through immunoblotting, and two clones, which expressed Abl comparable to that in parental Huh-7.5 cells, were obtained (WT#1 and WT#2) (Fig. 1*E*). Using pcDNA3-human editing-resistant (edR)-Abl-kinase-dead (KD), the same method as that used for establishing WT#1 and WT#2 cells was applied to obtain add-back cells stably expressing Abl-KD. Of the cell clones positive for Abl expression (Fig. 1*F*), two were selected for further study (KD#1 and KD#2). Sequence analysis confirmed that edR-Abl-WT or edR-Abl-KD complementary DNAs (cDNAs) were transcribed in these cells.

### The expression of Abl is involved neither in the process of HCV infection nor in the synthesis of viral protein

**Figure 2:**
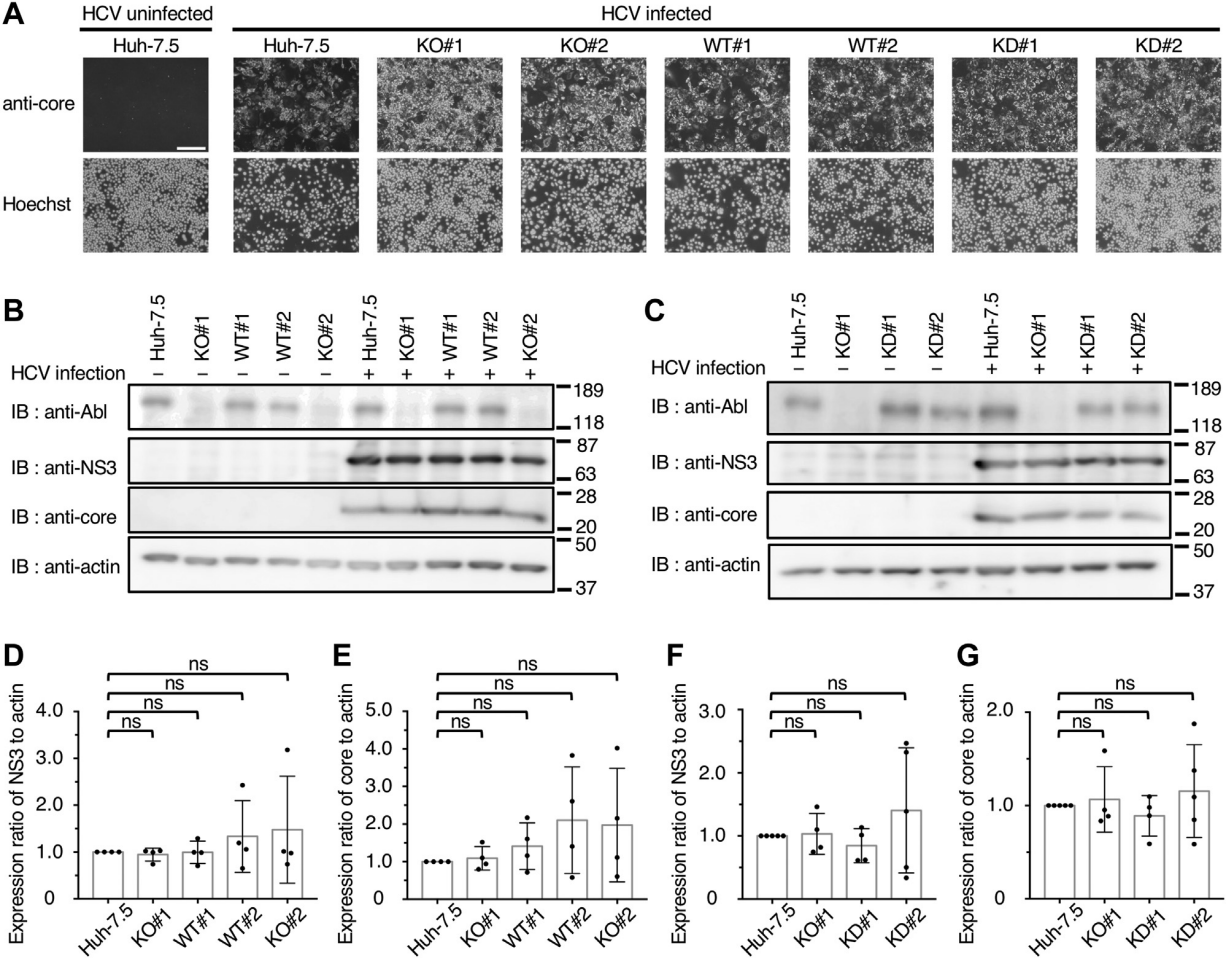
**Comparison of infection efficiency and viral protein synthesis between different cell lines.***A*, Huh-7.5 cells and Abl mutant cell lines were infected with HCVcc (J6/JFH1) at an MOI of 5. Then, fixed cells were stained with anticore mAb and Hoechst to visualize HCV core antigen (*upper panels*) and nuclei (*lower panels*), respectively. The scale bar represents 200 μm. *B*, the DSLs of Huh-7.5, Abl^−^ (KO#1), KO#1-derived Abl-WT/Abl^−^ (WT#1 and WT#2), and Abl^−^ (KO#2) cells were separated using SDS-PAGE and analyzed *via* immunoblotting with anti-Abl mAb, anti-NS3 antibodies, anticore mAb, and antiactin mAb. *C*, the DSLs of Huh-7.5, Abl^−^ (KO#1), and KO#1-derived Abl-KD/Abl^−^ (KD#1 and KD#2) cells were separated *via* SDS-PAGE and analyzed in the same way as shown in *B*. Molecular size markers are shown on the *right*. *D*–*G*, the expression ratios of NS3 (*D* and *F*) or core (*E* and *G*) to actin in each cell line were analyzed using ImageJ software and are presented as scatter plots with standard deviation. These results were obtained by four or five independent experiments for all cells. Abl, Abelson; DSL, detergent-soluble lysate; HCV, hepatitis C virus; HCVcc, cell culture-adapted HCV; KD, kinase-dead; mAb, monoclonal antibody; MOI, multiplicity of infection; NS3, nonstructural protein 3.

To investigate the effect of Abl expression on HCV life cycle, Huh-7.5, Abl^−^ (KO#1 and KO#2), Abl-WT/Abl^−^ (WT#1 and WT#2), and Abl-KD/Abl^−^ (KD#1 and KD#2) cells were infected with cell culture–adapted HCV (HCVcc) (29) at a multiplicity of infection of 5, and the infected cells were fluorescently stained using anticore monoclonal antibody (mAb) and Hoechst (Fig. 2*A*). The viral core antigen was detected in most cells in each cell line, suggesting that attachment and entry of HCV and translation of HCV proteins occur regardless of the expression or kinase activity of Abl. Then, intracellular accumulation levels of HCV proteins were compared among HCV-infected Huh-7.5, Abl^−^, Abl-WT/Abl^−^, and Abl-KD/Abl^−^ cells *via* immunoblotting (Fig. 2, *B* and *C*). A quantitative analysis indicated no significant differences in the expression levels of viral proteins NS3 (Fig. 2, *D* and *F*) and core (Fig. 2, *E* and *G*) in all infected cells. Furthermore, there was no significant difference in the amount of intracellular HCV RNA accumulated in HCV-infected Huh-7.5, Abl^−^, Abl-WT/Abl^−^, and Abl-KD/Abl^−^ cells (Fig. S1). These results suggest that Abl is not involved in viral gene expression and protein synthesis.

### Kinase activity of Abl is responsible for the assembly of HCV particles in Huh-7.5 cells

**Figure 3:**
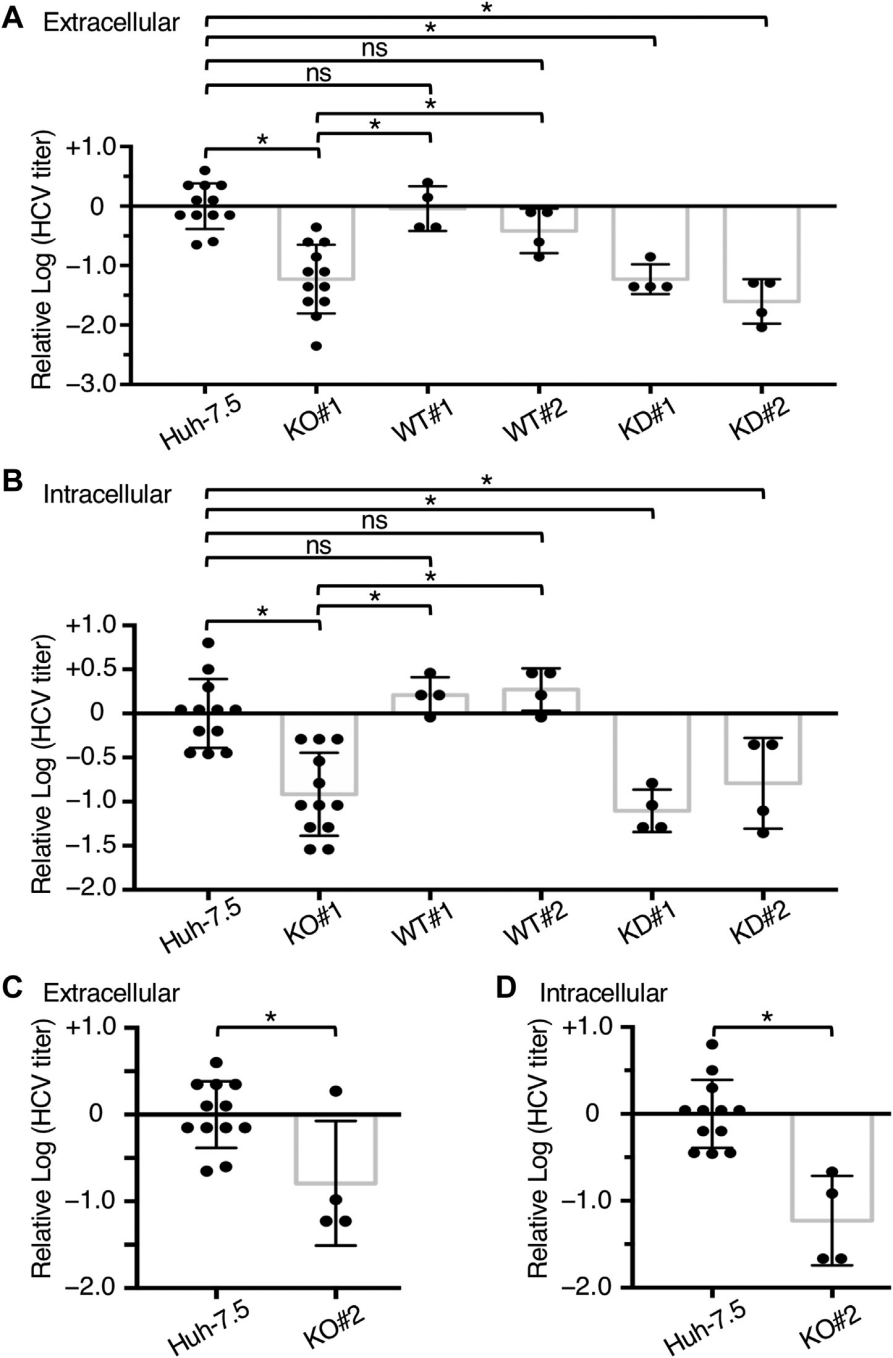
**Comparison of infectious particle production between different cell lines.** Dilution series of intracellular or extracellular virus suspensions recovered from each cell lines were prepared and inoculated into naive Huh-7.5 cells. After 72 h, cells were stained *via* immunofluorescence using the anticore mAb, and the TCID_50_ for each cell type was calculated. The results are from 4 to 12 independent experiments and presented as scatter plots with standard deviation. *Asterisks* indicate significant differences between paired values: ∗*p* < 0.05. The differences with *p* ≥ 0.05 were considered nonsignificant (*ns*). Comparison of extracellular (*A*) and intracellular (*B*) HCVs between Huh-7.5 cells with Abl^−^ (KO#1), Abl-WT/Abl^−^ (WT#1 and WT#2), and Abl-KD/Abl^−^ (KD#1 and KD#2) cells. Comparison of extracellular (*C*) and intracellular (*D*) HCVs between Huh-7.5 cells with Abl^−^ (KO#2) cells. Abl, Abelson; Abl^−^, Abl deficient; HCV, hepatitis C virus; KD, kinase-dead; mAb, monoclonal antibody; TCID_50_, median tissue culture infectious dose.

HCV particles are assembled through budding into the ER lumen and then released out of the cell. To evaluate whether Abl is involved in these steps of HCV infection, Huh-7.5, Abl^−^, Abl-WT/Abl^−^, and Abl-KD/Abl^−^ cells were infected with HCVcc, and extracellular and intracellular HCVs were titrated. The amounts of extracellular HCV released from KO#1 and KO#2 decreased significantly when compared with those from Huh-7.5 cells (Fig. 3, *A* and *C*). The amounts of intracellular HCV produced in infected KO#1 and KO#2 cells decreased to an order of magnitude (Fig. 3, *B* and *D*). These results suggest that Abl is involved in the assembly of infectious HCV particles into the ER lumen, as evidenced by the reduction in the amount of intracellular HCV, but not in the release of assembled HCV particles from the infected cell. In Abl^−^ cells generated using another method of transfection, large deletions and insertions were observed. This insertion sequence had an in-frame stop codon, and a decrease in the amount of intracellular viral particles was observed (Fig. S2).

Titers of infectious HCV were determined using the Abl-WT/Abl^−^ cell lines WT#1 and WT#2, which are descendants of KO#1 cells (Fig. 1*C*), and compared with the titers determined using Huh-7.5 cells and KO#1 cells. The amounts of extracellular HCV released from the two Abl-WT/Abl^−^ cell lines increased significantly when compared with those from KO#1 cells, whereas no significant difference was observed when compared with the amounts of extracellular HCV released from Huh-7.5 cells (Fig. 3*A*). Similar results were obtained regarding the amounts of intracellular HCV (Fig. 3*B*). These findings suggest that the decreased capacity of KO#1 cells to produce HCV (Fig. 3*A*) is caused by mutations in *abl* and not by the off-target effects of genome editing.

Furthermore, infectious HCV titers were determined using the Abl-KD/Abl^−^ cell lines KD#1 and KD#2, which are descendants of KO#1 cells (Fig. 1*C*), and compared with the titers determined using Huh-7.5 cells and KO#1 cells. The capacity of KD#1 and KD#2 cells to produce HCV was not restored to the same level as that of Huh-7.5 cells, unlike those of WT#1 and WT#2 cells (Fig. 3, *A* and *B*), suggesting that the kinase activity of Abl is necessary for the formation of HCV particles in Huh 7.5 cells.

### Reconstitution experiments revealed that NS5A acts as an activator of Abl

**Figure 4:**
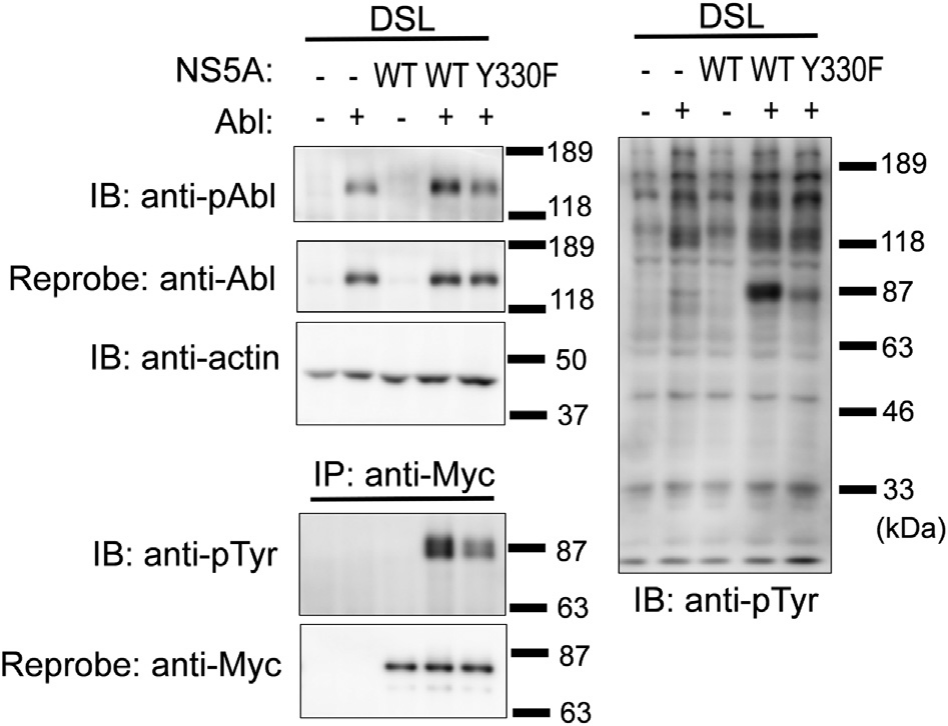
**Activation of Abl by tyrosine phosphorylation of NS5A.** HEK 293T cells were transiently transfected with a combination of pcDNA3-Abl-WT, pEF1-NS5A-WT/myc-His A, or pEF1-NS5A-Y330F/myc-His A. The DSL was separated using SDS-PAGE and analyzed *via* immunoblotting with anti-phospho-Abl (anti-pAbl), anti-Abl, antiactin, and anti-pTyr mAbs. Molecular size markers are shown on the *right*. Abl, Abelson; DSL, detergent-soluble lysate; HEK 293T, human embryonic kidney 293T cell line; mAb, monoclonal antibody; NS5A, nonstructural protein 5A.

To evaluate the interaction between NS5A and Abl in more detail, human Abl and JFH1 strain–derived NS5A were coexpressed using HEK 293T cells. We found that phosphorylation of Abl on Tyr^412^ was enhanced upon coexpression with NS5A-WT (Fig. 4, *fourth lane* in the *left upper panel*), although lower phosphorylation of Abl was observed in the lysate prepared from cells transfected with Abl cDNA alone (Fig. 4, *second lane* in the *left upper panel*); this suggests that NS5A acts as an activator of Abl, because Tyr^412^ is located in the activation loop of the kinase domain of Abl, and its phosphorylation leads to the catalytic activation to Abl (30, 31). Notably, no enhancement of Tyr^412^ phosphorylation of Abl was observed when Abl was coexpressed with NS5A-Y330F (Fig. 4, *fifth lane* in the *left upper panel*). These results suggest that the tyrosine-phosphorylated NS5A acts as an activator of Abl.

Tyrosine phosphorylation of NS5A was evaluated *via* immunoprecipitation with the anti-Myc mAb and immunoblotting with the antiphosphotyrosine (pTyr) mAb. As observed in a previous study using NS5A (Con1) and mouse Abl (25), NS5A-WT (JFH1) was tyrosine phosphorylated by human Abl, whereas NS5A-Y330F was tyrosine phosphorylated at a relatively lower level (Fig. 4, *left lower panel*). Notably, no increase in the Tyr^412^ phosphorylation of Abl was observed when Abl was coexpressed with NS5A-Y330F (Fig. 4, *left lower panel*).

Tyrosine phosphorylation of cellular proteins was analyzed *via* immunoblotting with the anti-pTyr mAb (Fig. 4, *right panel*). The detected proteins were classified into two groups. The first group contained protein tyrosine phosphorylated *via* transfection with Abl cDNA alone, most of which had molecular weights of ≥118 kDa. The phosphorylation levels of these proteins barely increased after coexpression with NS5A. The second group contained proteins phosphorylated remarkably after coexpression with NS5A. Proteins with molecular weights of approximately 87 kDa were mainly detected and speculated to be tyrosine-phosphorylated NS5A, as assessed from immunoprecipitation results shown in the *lower left panel* of Figure 4.

### Abl forms a complex with NS5A through tyrosine phosphorylation events

**Figure 5:**
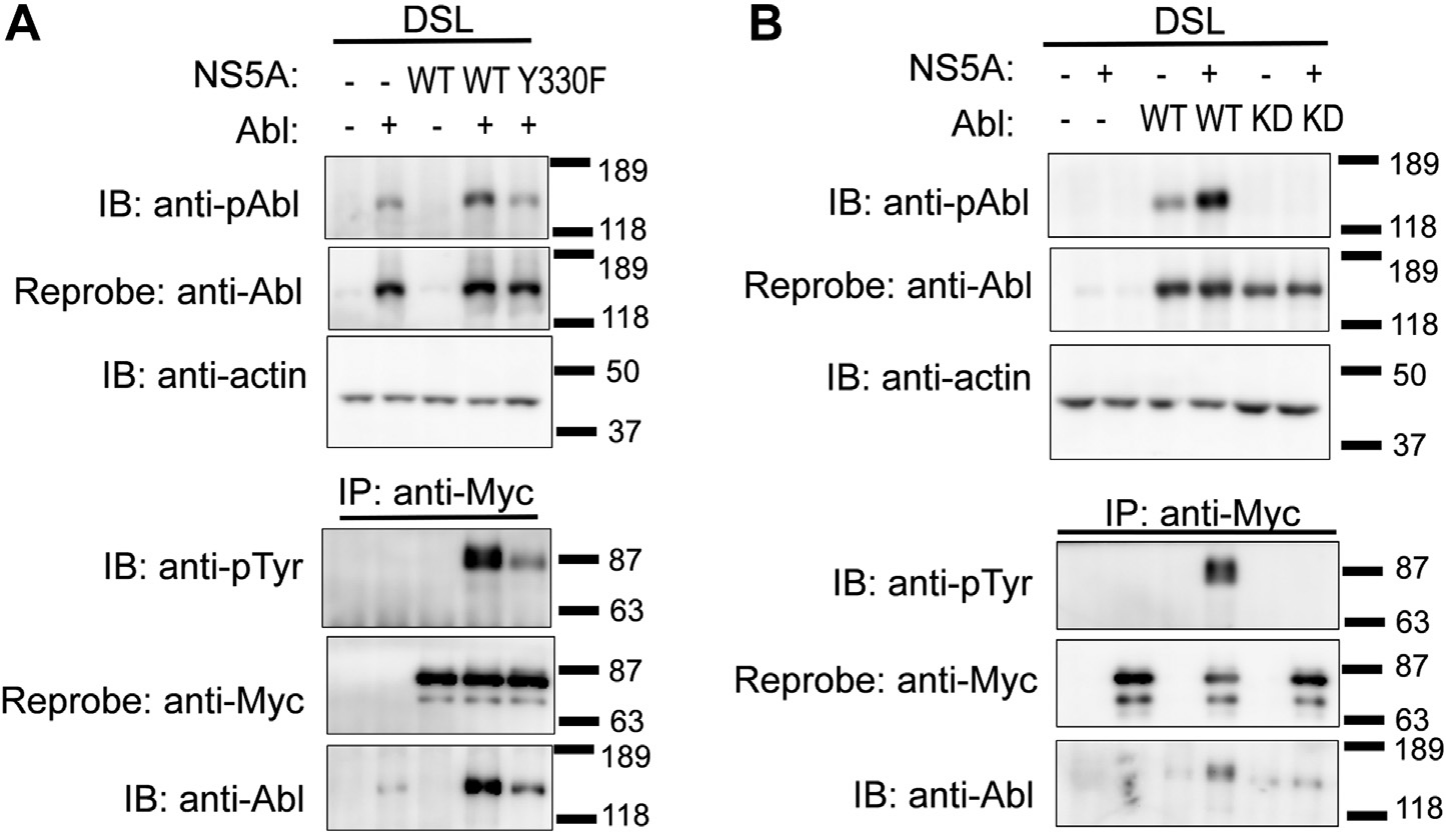
**Association of Abl with tyrosine-phosphorylated NS5A.***A* and *B*, HEK 293T cells were transiently transfected with a combination of pcDNA3-Abl-WT, pcDNA3-Abl-KD, or pEF1-NS5A-WT/myc-His A. Cells were lysed in lysis buffer lacking DOC and SDS. DSL and anti-Myc immunoprecipitates were separated using SDS-PAGE and analyzed *via* immunoblotting with the indicated antibodies. Molecular size markers are shown on the *right*. Abl, Abelson; DOC, sodium deoxycholate; DSL, detergent-soluble lysate; HEK 293T, human embryonic kidney 293T cell line; KD, kinase-dead; NS5A, nonstructural protein 5A.

Previously, we showed that NS5A binds to the SH3 domain of Abl using a pull-down assay (32), which suggested that NS5A forms a complex with Abl. To evaluate whether Abl interacts with NS5A, both were coexpressed in HEK 293T cells. Cells were lysed in lysis buffer lacking sodium deoxycholate (DOC) and SDS, which can disrupt the physical association between Abl and NS5A. The results from immunoblotting using detergent-soluble lysate (DSL) (Fig. 5*A*, *left upper panels*) were the same as observed in Figure 4, indicating that Abl and NS5A were solubilized appropriately. The immunoprecipitation experiment revealed that Abl interacted with Myc-tagged NS5A, and this association was decreased when Abl was coexpressed with Myc-tagged NS5A-Y330F (Fig. 5*A*, *left lower panels*). Control experiments using the anti-Stat5 mAb, of the same subclass immunoglobulin G1 (IgG1) as the anti-Myc antibody, failed to detect sufficient Abl in the immunoprecipitates (Fig. S3). In addition, Abl-KD failed to coimmunoprecipitate with NS5A-WT (Fig. 5*B*). These findings suggest that Abl forms a complex with NS5A through the tyrosine phosphorylation of NS5A by Abl. The kinase activity of c-Abl was required for complex formation between Abl and NS5A.

### Abl forms a complex with monomeric NS5A regardless of tyrosine phosphorylation events

**Figure 6:**
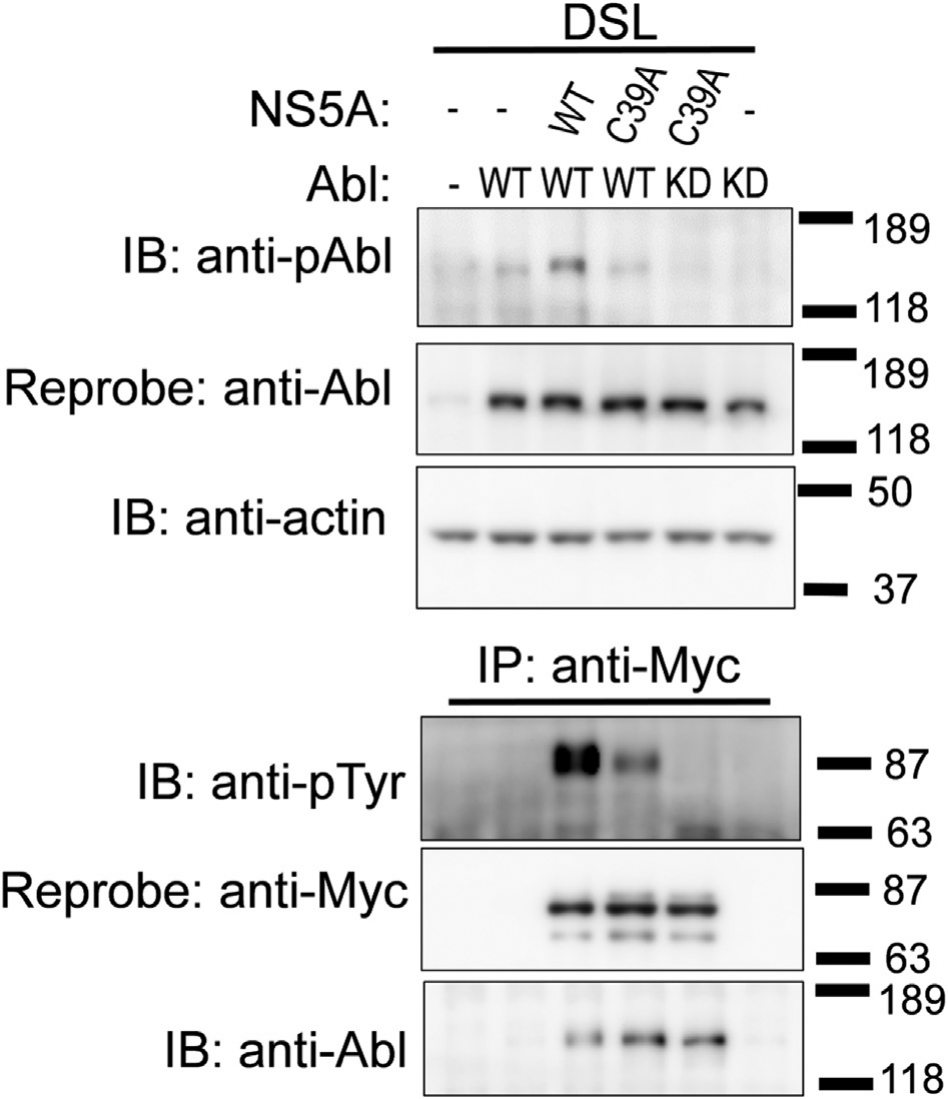
**Analysis of the association between monomeric NS5A and Abl.** HEK 293T cells were transiently transfected with a combination of pcDNA3-Abl-WT, pcDNA3-Abl-KD, pEF1-NS5A-WT/myc-His A, or pEF1-NS5A-C39A/myc-His A. The DSL was separated *via* SDS-PAGE and analyzed through immunoblotting with anti-phospho-c-Abl (anti-pAbl), anti-Abl, antiactin, anti-pTyr, and anti-Myc mAbs. Molecular size markers are shown on the *right*. Abl, Abelson; DSL, detergent-soluble lysate; HEK 293T, human embryonic kidney 293T cell line; KD, kinase-dead; mAb, monoclonal antibody; NS5A, nonstructural protein 5A.

To evaluate the effects of C39A mutation on the NS5A-mediated activation of Abl and the association of NS5A with Abl, NS5A-WT or NS5A-C39A was coexpressed with Abl-WT or Abl-KD in HEK 293T cells. The NS5A-mediated tyrosine phosphorylation of Abl on Tyr^412^ decreased when Abl was expressed with NS5A-C39A, suggesting that homodimerization is required for NS5A to activate Abl (Fig. 6, *upper panel*). Simultaneously, the Abl-mediated tyrosine phosphorylation of NS5A also decreased because of the C39A mutation (Fig. 6, *lower panel*). Even though the tyrosine phosphorylation events of both Abl and NS5A were not promoted by the C39A mutation, the association of Abl with NS5A-C39A was comparable with that of NS5A-WT (Fig. 6, *lower panel*). Unlike NS5A-WT, NS5A-C39A was coimmunoprecipitated with Abl-KD as well as Abl-WT (Fig. 5*B* *versus* Fig. 6). These results suggest the existence of another mode of complex formation between Abl and monomeric NS5A, which occurs independently of tyrosine phosphorylation events.

## Discussion

Viral proteins encoded by the HCV genome interact with various human host factors, some of which regulate the HCV life cycle and are involved in the development of pathogenicity. We found that administration of imatinib reduced viral particle production during the HCV life cycle in cultured hepatocytes infected with HCV (J6/JFH1). Our study is the first to demonstrate that the protein tyrosine kinase activity of Abl is involved in the molecular mechanism underlying this process.

In this study, we established Abl^−^ cells and Abl^−^ cells expressing WT or KD Abl and compared the HCV production ability of these cells with parental Huh-7.5 cells. We found that the expression of Abl with kinase activity promoted virus production (Figure 1, Figure 2, Figure 3). To our knowledge, only a few reports of viruses that utilize AFK during virus particle production or life cycle exist (33). In cells infected with the pox virus, the step of releasing enveloped virus bound to the cell surface into the culture supernatant is facilitated by Abl/Arg (26). In cells infected with the Ebola virus, Abl contributes to the process of “horizontal” budding and release of fibrous virus particles from the cell membrane (27, 34). By contrast, Abl promotes the formation of HCV particles (Figure 1, Figure 2, Figure 3). Notably, HCV particle formation occurs *via* budding from the ER membrane (1, 3). This mechanism of virus production differs from that of pox virus and Ebola virus, which release the virus from the cytoplasmic membrane. Accordingly, we aimed to establish whether the assembly of HCV particles is directly promoted by Abl.

**Figure 7:**
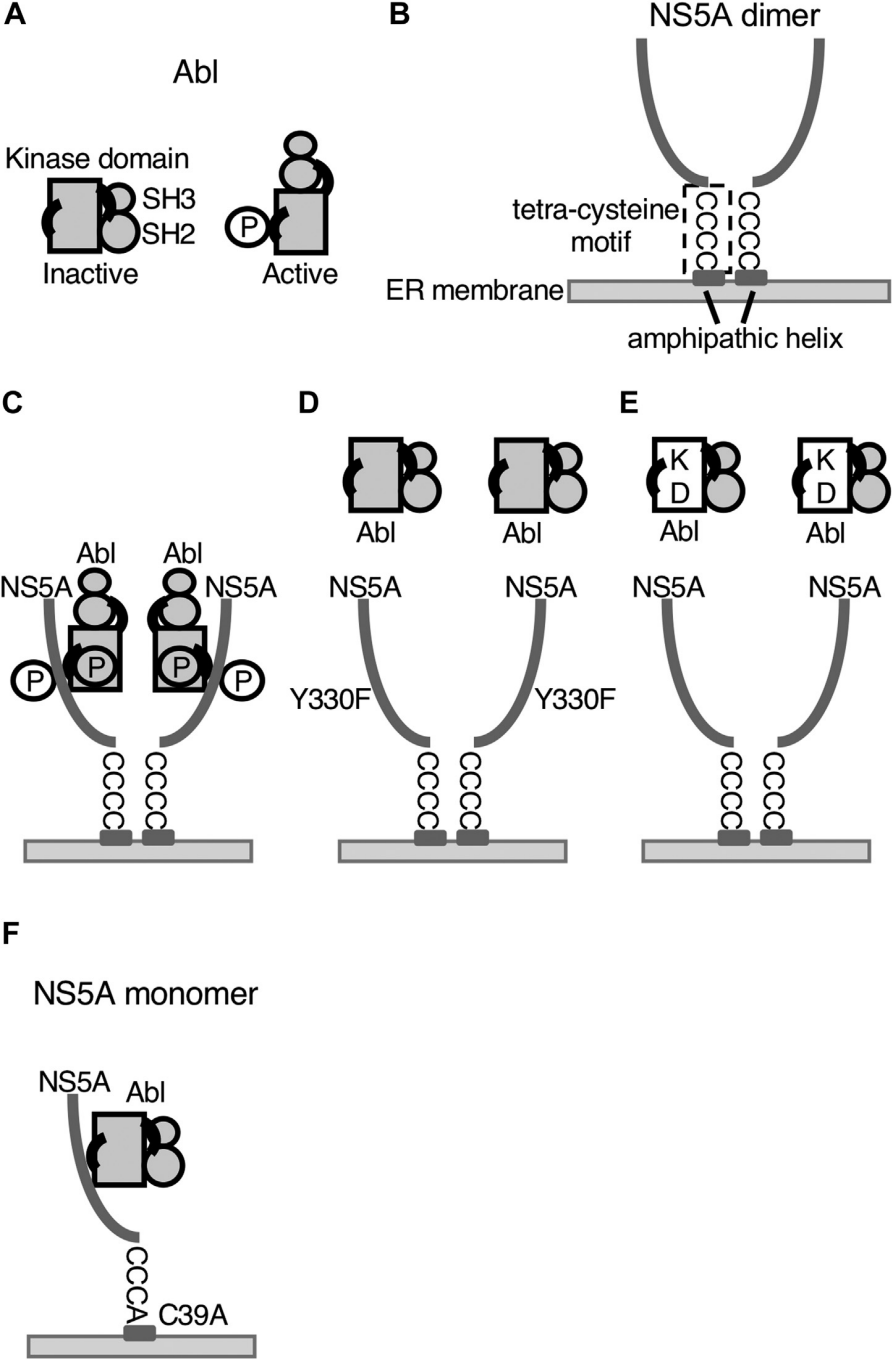
**Possible model of the association between Abl and NS5A.***A*, structural motif of Abl. “Clamped” conformation as an inactive state (22) and “unclamped” conformation as an active state with tyrosine phosphorylation at Tyr^412^ in the activation loop of the kinase domain (*right*). *B*, structural motifs of the NS5A dimer. Amphiphilic helix and four cysteine residues (Cys^39^, Cys^57^, Cys^59^, and Cys^80^) are involved in NS5A dimerization on the ER membrane. *C*–*F*, comparison of the association of Abl with NS5A. Abl-WT and NS5A-WT (*C*). Dimerization of NS5A, formation of the NS5A–Abl complex, and phosphorylation of NS5A and Abl are induced. Abl-WT and NS5A-Y330F (*D*) and Abl-KD and NS5A-WT (*E*). Dimerization of NS5A has occurred; however, neither formation of the NS5A–Abl complex nor the tyrosine phosphorylation of NS5A or Abl is induced. Abl-WT and NS5A C39A (*F*). Formation of the NS5A–Abl complex has occurred; however, neither the dimerization of NS5A nor the tyrosine phosphorylation of NS5A or Abl is induced. Abl, Abelson; ER, endoplasmic reticulum; KD, kinase-dead; NS5A, nonstructural protein 5A.

Reconstitution experiments revealed that NS5A acts as an activator of Abl (Figs. 4 and 5). Abl is activated by the phosphorylation of Tyr^412^ in the activation loop (Fig. 7*A*) (30, 31). Abl forms a complex with NS5A through tyrosine phosphorylation events (Fig. 5). These findings suggest that NS5A can also function as an activator of Abl by serving as a substrate for Abl. Abl is activated by substrates such as Abi and Crk (24), and NS5A may function through a similar mechanism. Consistently, the Y330F mutation in NS5A disrupted this activator function of NS5A, with a decrease in the tyrosine phosphorylation of NS5A by Abl (Figs. 4 and 5). This result is consistent with the findings obtained in our previous study that the Y330F mutation reduces both the tyrosine phosphorylation level of NS5A and the efficiency of HCV particle assembly in HCV-infected Huh-7.5 cells (25), suggesting that the substrate–activator function of NS5A is valid even in the context of virus infection.

In addition, our findings revealed that homodimerization of NS5A is required for Abl activation (Fig. 6). NS5A is anchored to the ER membrane by an amphipathic helix at its N terminus and homodimerizes through a tetracysteine motif next to the helix (Fig. 7*B*). These findings are reminiscent of canonical signaling pathways wherein receptor dimerization occurs because of interaction with extracellular signals, leading to the reciprocal phosphorylation of tyrosine kinases associated with or located in the cytoplasmic domain of the receptor (35).

Under conditions where NS5A and Abl are phosphorylated, the association of both was observed. However, the formation of complexes containing NS5A and Abl was scarcely observed when NS5A or Abl was mutated to inhibit the phosphorylation event under the conditions where homodimerization of NS5A was possible (Figure 4, Figure 5, Figure 6 and 7, *C*–*E*). In addition, NS5A with a dimerization-defective mutation lost its ability to activate Abl but could only interact with Abl (Figs. 6 and 7*F*), suggesting that homodimerization of NS5A was the driving force for Abl activation. Although the activation mechanism of Abl is structurally similar to that of its close relative Src, it has been reported that Abl is activated by oligomerization, perhaps involving regulation by an interaction partner, in the overexpression system using COS cells (36). Moreover, in the case of BCR-Abl, the fused BCR associates with the Abl-SH2 domain in a non–pTyr-dependent manner (37, 38). Therefore, the possibility of NS5A C39A dimerization in association with Abl cannot be completely ruled out. Further extensive biophysical studies are required to validate the establishment of the stoichiometry of this complex.

A common feature among the pox virus, Ebola virus, and HCV in their interactions with Abl is that viral proteins are tyrosine phosphorylated by AFKs. The membrane protein A36R of vaccinia virus, a type of pox virus, is tyrosine phosphorylated not only by Src family kinases but also by AFKs (39, 40). Phosphorylated A36R recruits NCK1 and N-WASP (39); this recruitment is thought to stimulate Arp2/3-mediated actin polymerization (41, 42). By contrast, the structural protein VP40 of the Ebola virus (27) and the NS5A of HCV are tyrosine phosphorylated by Abl. However, the mechanisms downstream of VP40 and NS5A tyrosine phosphorylation are unclear.

Pox virus, Ebola virus, and HCV produce virus particles through a unique mechanism, and there seems to be no similarity among them except that they are envelope positive. Therefore, it is difficult to predict whether envelope-positive viruses other than these three utilize Abl in the virus production step. Our findings suggest that NS5A recruits and activates Abl on the ER membrane during the production of viral particles. By drawing an analogy with the A36R protein of vaccinia virus, we speculate that the tyrosine-phosphorylated NS5A recruits other Abl substrates. We selected hepatocyte growth factor–regulated tyrosine kinase substrate, which has been reported to be involved in HCV particle formation (43, 44), as a candidate of Abl substrate and evaluated hepatocyte growth factor–regulated tyrosine kinase substrate phosphorylation in HCV-infected cells through immunoblotting. Unfortunately, we did not detect them (Fig. S4). In the future, indiscriminate and cross-sectional studies on various viruses to determine the general mechanism of Abl utilization in the virus production step are warranted. In such studies, Abl^−^ cells and Abl-add-back cells (Abl-WT/Abl^−^ cells or Abl-KD/Abl^−^ cells) will be useful.

Recent study reported the effects of Abl on HCV life cycle; Min *et al.* (45) have observed that Abl knockdown significantly decreased HCV RNA and protein expression levels in HCV-infected cells, speculating that Abl is required for HCV entry. In addition, HCV pseudoparticle infection assays have shown that Abl is involved in HCV entry at the clathrin-mediated endocytosis stage. However, in the present study, Abl was found to be a requirement for HCV particle formation. Furthermore, Abl expression was observed to be associated neither with the HCV infection process nor with the viral protein synthesis (Fig. 2). The difference in the experimental systems utilized in both studies may have caused the discrepancy in the results. Therefore, the involvement of Abl in viral entry cannot be entirely dismissed, and the mechanism through which Abl affects the various host factors involved in viral HCV particle formation remains to be investigated in future studies.

The current treatment for HCV infection has considerably improved because of the advent of direct-acting antivirals (DAAs), which act directly on viral proteins. However, new challenges have arisen, such as the need to overcome intractable cases because of the emergence of DAA-resistant viruses, the pathogenic mechanism of liver cancer after virus elimination, and underlying diseases. Sufficient clinical knowledge has already been accumulated on the safety of protein tyrosine kinase inhibitors. Since the mechanism of action is different from that of existing therapeutic agents, it is expected that it may be effective against DAA-resistant viruses. Overcoming hepatitis C is an important social issue that will contribute to the improvement of public health and medical care. In addition, it is expected that our results will provide reference for advanced study on the development of effective therapeutic strategies in preparation for emerging infectious diseases caused by Flaviviridae viruses closely related to HCV.

## Experimental procedures

### Cell culture and generation of Abl-deficient cells

Huh-7.5 cells were kindly provided by Dr Charles M. Rice (Rockefeller University). Huh-7.5 and HEK 293T cells were cultured in Dulbecco’s modified Eagle’s medium supplemented with 0.1 mM nonessential amino acids (Wako Chemicals) and 10% fetal bovine serum (Sigma–Aldrich). To establish Abl-deficient (Abl^−^) cells, a plasmid expressing Cas9 and guide RNA targeting exon 2 of *abl* (46) was cotransfected into Huh-7.5 cells with pcDNA3.1(−) (Thermo Fisher Scientific), which contains G418-resistant *neoR*. Selection with G418 was performed, and drug-resistant clones were obtained. Expression of Abl was analyzed *via* immunoblotting to identify Abl^−^ clones. Sequence analysis confirmed one mutation type in KO#1 cells and two mutation types (alleles 1 and 2) in KO#2 cells, indicating the distinct nature of the two strains (Fig. 1*B*). Although it is not possible to determine the number of alleles of the *abl* gene from this result, analysis of the subcloned DNA fragments (13 for KO#1 and 11 for KO#2) did not confirm the presence of the third mutation. This finding revealed that Huh-7.5 cells possess two or, less likely, more than two alleles for the *abl* gene, and the results from the direct DNA sequence analysis performed prior to subcloning corroborated this (Fig. S5). These mutations caused frameshift in the *abl* gene reading frame and abortive terminations of translation within exon 2. The termination codon was observed to be inserted at 42 (KO#1 alleles 1 and 2 and KO#2 allele 1) and 102 (KO#2 allele 2) base pairs after the mutation sites (Fig. S6).

### Antibodies

Anti-c-Abl mAb (8E9) (catalog number: sc-56887; lot number: C2117), anti-β-actin mAb (C4) (catalog number: sc-47778; lot number: A2017), and anti-Myc/c-Myc mAb (9E10) (catalog number: sc-40; lot number: A2814) were purchased from Santa Cruz Biotechnology. Anti-HCV Core 1b mAb (clone C7-50) (catalog number: ab2740; lot number: GR3249588-2) was obtained from Abcam. Anti-mouse IgG (H+L), F(ab')2 fragment (Alexa Fluor 488 conjugate) (catalog number: 4408; lot number: 18), and anti-phospho-c-Abl (Tyr412) (247C7) (catalog number: 2865; lot number: 3) mAb were purchased from Cell Signaling Technology. Anti-HCV NS3 protein polyclonal antibodies (catalog number: GTX131276; lot number: 41836) were obtained from GeneTex. Anti-pTyr mAb (4G10) (catalog number: 05-321; lot number: 2138006) was obtained from Sigma–Aldrich.

### Plasmids

Primers used in this study are listed in Table 1. pcDNA3-human Abl, a plasmid DNA for expressing variant b of human Abl, has been described previously (25). For add-back expression of Abl-WT in Abl^−^ cells, the edR form (edR-Abl-WT), which contains two synonymous mutations in the target sequence (*abl*), was generated through site-directed mutagenesis (QuikChange Site-Directed Mutagenesis Kit; Agilent Technologies), using primers 1 and 2. The KD forms of Abl (Abl-KD and edR-Abl-KD) were generated *via* the site-directed mutagenesis of Lys^290^ to Met using primers 3 and 4. The resulting cDNAs were sequenced to confirm that mutations were introduced only in the target sites (Fig. 1*B*).

**Table 1:** Primer sequences used in this study

Primer number	Primer sequence (5′ to 3′)
1	GTCTGAGTGAAGCCGCCCGCTGGAACTCCAAGGAAA
2	TTTCCTTGGAGTTCCAGCGGGCGGCTTCACTCAGAC
3	GACGGTGGCCGTGATGACCTTGAAGGAGG
4	CCTCCTTCAAGGTCAACACGGCCACCGTC
5	GGGGTACCATGTCCGGATCCTGGCTCC
6	GGTCTAGAGCAGCACACGGTGGTATC
7	CGGCCTCCCCTTCATCTCTGCTCAAAAGGGGTACA
8	TGTCCCCTTTTGAGCAGAGATGAAGGGGAGGCCG

NS5A cDNAs from pSGR-JFH1 (47) and pSGR-JFH1 (Y330F) (25) were subcloned into the pEF1/myc-His A vector (Thermo Fisher Scientific) using primers 5 and 6 to create pEF1-NS5A-WT/myc-His A and pEF1-NS5A-Y330F/myc-His A, respectively. pEF1-NS5A-C39A/myc-His A was generated *via* the site-directed mutagenesis of Cys^39^ to Ala using primers 7 and 8. The resulting cDNAs were sequenced to confirm that mutations were introduced only in the target sites (Fig. 1*C*).

### Stable add-back expression of Abl in Abl^−^ cells

To establish Abl-add-back (Abl-WT/Abl^−^ or Abl-KD/Abl^−^) cells, pcDNA3-edR-Abl-WT or pcDNA3-edR-Abl-KD was cotransfected with pENTR-BSR, which contains the blasticidin-resistant *bsr* (a gift from Dr Tomohiro Kurosaki, Osaka University, Japan), into an Abl^−^ clone 1 (KO#1). Blasticidin selection was performed to obtain resistant clones, and the expression of edR Abl proteins was evaluated *via* immunoblotting.

### HCV infection and titer determination

HCVcc (29), which was derived from the J6/JFH1 chimeric strain (48) and kindly provided by Dr Charles M. Rice, was further passaged in Huh-7.5 cells to prepare virus stocks. Dilutions of the supernatants of infected cell cultures were inoculated into naïve Huh-7.5 cells. Seventy-two hours after infection, cells were analyzed *via* immunostaining using the anti-HCV core mAb to identify core-positive cells, and median tissue culture infectious doses (TCIDs_50_) were determined.

Huh-7.5 cells were inoculated with virus stocks at a multiplicity of infection of 5 (TCID_50_/cell). Twenty-four hours after infection, the medium was replaced with fresh medium. For preparation of extracellular HCV samples, culture supernatants were collected after 72 h of infection. For preparing intracellular HCV samples, infected cells were stripped and suspended in distilled water. After three cycles of freezing and thawing, the cell suspensions were centrifuged at 2000*g* for 2 min, and the supernatants were collected. The TCID_50_ values of the collected extracellular or intracellular samples were determined as described above.

### Immunostaining

HCV-infected cells were fixed in cold MeOH (−20 °C) for 10 min and blocked with the TNB blocking buffer (PerkinElmer) for 1 h. Cells were incubated with the anti-HCV core mAb overnight at 4 °C. Anti-mouse IgG (H+L), F(ab')2 fragment (Alexa Fluor 488 conjugate) was used as the secondary antibody. Nuclei were stained with Hoechst 33258 (Cellstain; Dojindo Laboratories). Cells were analyzed with an inverted fluorescence microscope (Olympus IX71; Olympus).

### Transient transfection

For transient coexpression experiments, HEK 293T cells were cotransfected with the combinations of pcDNA3-Abl-WT or pcDNA-Abl-KD and pEF1-NS5A-WT/myc-His A, pEF1-NS5A-Y330F/myc-His A, or pEF1-NS5A-C39A/myc-His A using a nonlipid-based chemical transfection reagent (GeneJuice Transfection Reagent; Sigma–Aldrich). After 22 h of transfection, the cells were used for immunoprecipitation and immunoblotting.

### Immunoprecipitation and immunoblotting

Cells were lysed in lysis buffer (50 mM Tris [pH 7.4], 150 mM NaCl, 10 mM EDTA, 100 mM NaF, 1 mM Na_3_VO_4_, 1% Triton X-100, 1 mM PMSF, and 2 μg/ml aprotinin) containing 0.5% DOC and 0.1% SDS, and then centrifuged at 17,800*g* and 4 °C for 10 min to obtain the DSL. For immunoprecipitation, the DSL was first incubated overnight with the anti-Myc mAb at 4 °C and then with the rabbit anti-mouse antibody (Jackson ImmunoResearch Laboratories) (catalog number: 315-005-045; lot number: 67802) for 2 h at 4 °C. Then, the lysate was incubated with protein A-Sepharose (Sigma–Aldrich) beads for 1 h at 4 °C. The immunoprecipitates were collected by centrifugation, and the beads were washed thrice with lysis buffer. The immunoprecipitated proteins were eluted through heat treatment for 5 min at 100 °C with 2× sampling buffer.

The DSL and immunoprecipitated proteins were separated using SDS-PAGE and transferred onto Immobilon-P Transfer Membranes (Merck Millipore). The membranes were blocked in 25 mM Tris, pH 8.0, 150 mM NaCl, and 0.1% Tween-20 buffer containing 5% nonfat dried milk and incubated first with primary antibodies and then with secondary antibodies (peroxidase-conjugated AffiniPure Goat Anti-Mouse IgG [H+L] [catalog number: 115-035-146; lot number: 72985] or peroxidase-conjugated AffiniPure Goat Anti-Rabbit IgG [H+L] [catalog number: 111-035-003; lot number: 76254], Jackson ImmunoResearch Laboratories). The chemiluminescent bands were visualized using an enhanced chemiluminescence reagent (Western Lightning; PerkinElmer Life Sciences) and LAS-3000 mini (Fujifilm). To analyze protein–protein interactions, cells were lysed in lysis buffer lacking DOC and SDS.

To indicate the positions of molecular weight markers above and below the bands of interest, two types of molecular weight markers, one from Bio-Rad (catalog number: 161-0373; batch number: 64396572) and the other from Nacalai (catalog number: 02525; lot number: L8P6952), were used. The expression levels of the viral proteins were quantified as follows: the median density of the bands corresponding to NS3, core, and actin obtained by immunoblotting was determined using ImageJ bundled with Java 1.8.0_172 (National Institutes of Health). The median density of NS3 and core was divided by the median density of each actin, and comparisons were made among clones using Huh-7.5 cells as a reference (n = 4 or 5).

### Statistical analysis

A Mann–Whitney *U* test (unpaired test) was performed to determine the significance of the differences between paired values shown in Figure 2. Student’s *t* test (unpaired test) was performed to determine the significance of differences between paired values shown in Figure 3 and Fig. S1. *p* Values less than 0.05 were considered significant.

## Data availability

All data are contained within the article.

## Supporting information

This article contains supporting information (25).

## Conflict of interest

The authors declare that they have no conflicts of interest with the contents of this article.
