# Supplementary material for: Protein tyrosine kinase Abl promotes hepatitis C virus particle assembly via interaction with viral substrate activator NS5A
Source: J Biol Chem. 2022 Mar 4;298(4):101804. doi: 10.1016/j.jbc.2022.101804 (PMC8980994; doi:10.1016/j.jbc.2022.101804)
Supplement: Supplemental Figures S1–S6 [file mmc1.docx]

JBC-D-21-00486R3

**Protein tyrosine kinase Abl promotes hepatitis C virus particle assembly via interaction with viral substrate activator NS5A**

Daisuke Miyamoto, Kenji Takeuchi, Kazuyasu Chihara, Shigeharu Fujieda, and Kiyonao Sada

**Supporting Material List**

**Figure S1. Comparison of intracellular HCV RNA accumulated in HCV-infected Huh-7.5, Abl^−^, Abl-WT/Abl^−^, and Abl-KD/Abl^−^ cells.**

**Figure S2. Analysis of Abl^−^ cells generated using another method of transfection.**

**Figure S3. Control experiments using anti-Stat5 mAb.**

**Figure S4. Analysis of HRS phosphorylation in HCV-infected cells.**

**Figure S5. Direct DNA sequence analysis of the *abl* gene in Abl^−^ cells.**

**Figure S6. In-frame termination codons generated by genome editing in the *abl* gene of Abl^−^ cells.**


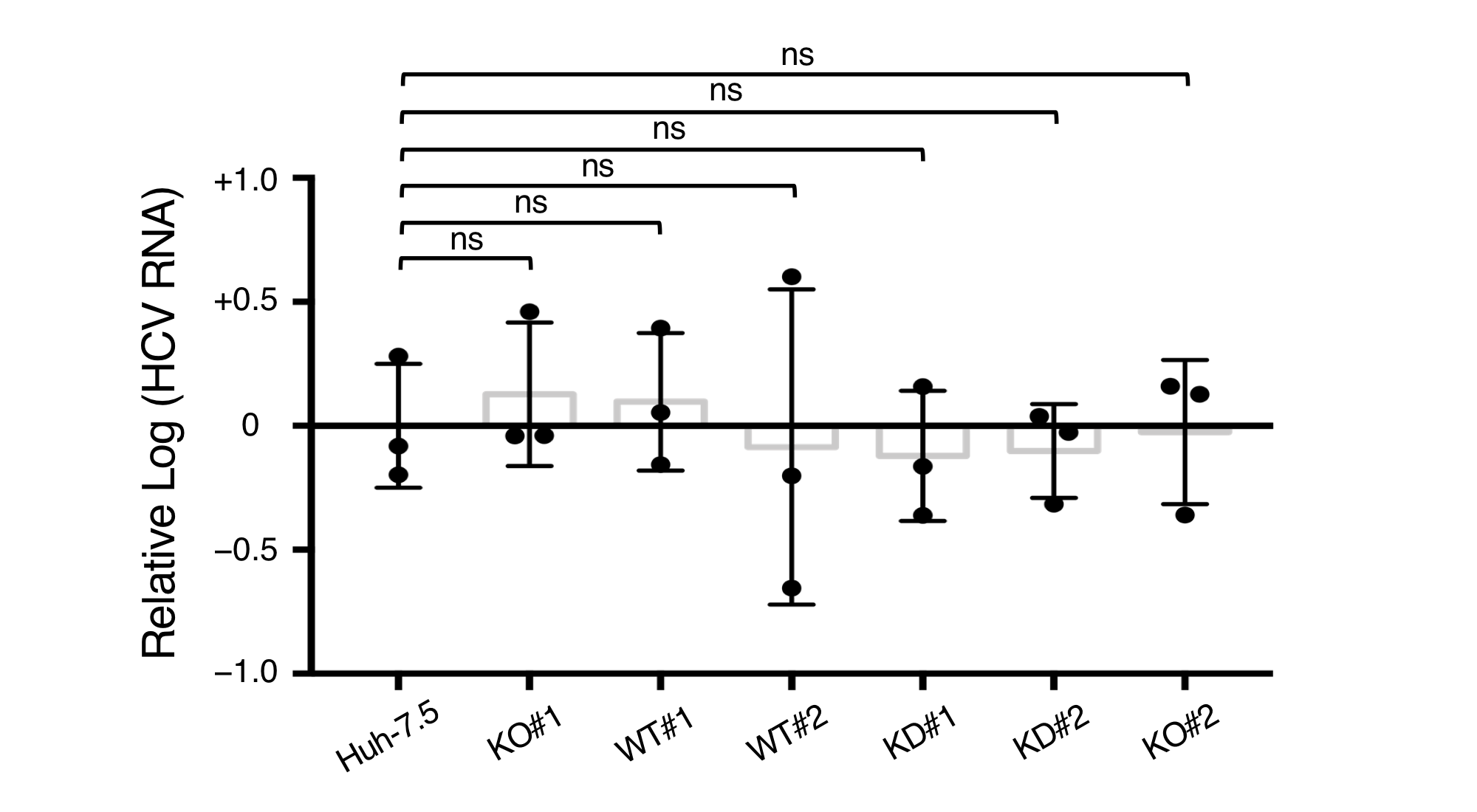


**Figure S1. Comparison of intracellular HCV RNA accumulated in HCV-infected Huh-7.5, Abl^−^, Abl-WT/Abl^−^, and Abl-KD/Abl^−^ cells.**

Intracellular HCV RNA was quantified by real-time PCR and normalized to control values. Real-time PCR was performed as described previously (Yamauchi *et al*. 2015). Cells were infected with HCVcc (J6/JFH1) at an MOI of 5. At 72 h post-infection, total RNA was prepared using High Pure RNA isolation kit (Roche, Mannheim, Germany). RNA was reverse-transcribed using RevertraAce qPCR RT Master Mix with gDNA Remover (Toyobo, Osaka, Japan). Real-time PCR was performed using a SYBR FAST qPCR kit (KAPA Biosystems, MA, USA) and StepOne Plus real-time PCR system (Life Technologies, CA, USA). PCR primers for amplification of HCV cDNA were described previously (Yamauchi *et al*. 2015). To construct standard curves for HCV RNA, total RNA derived from HCV-infected Huh 7.5 cells was used as a template for normalization within each experiment, and *in vitro* transcribed HCV RNA was used for normalization among experiments. Bars represent the relative logarithmic mean +/- standard deviation (n=3). *, p<0.05. The differences with P ≥0.05 were considered non-significant (*ns*).

Abl: Abelson, Abl^−^: Abl-deficient, HCV: hepatitis C virus, HCVcc: cell culture-adapted HCV, KD: kinase-dead


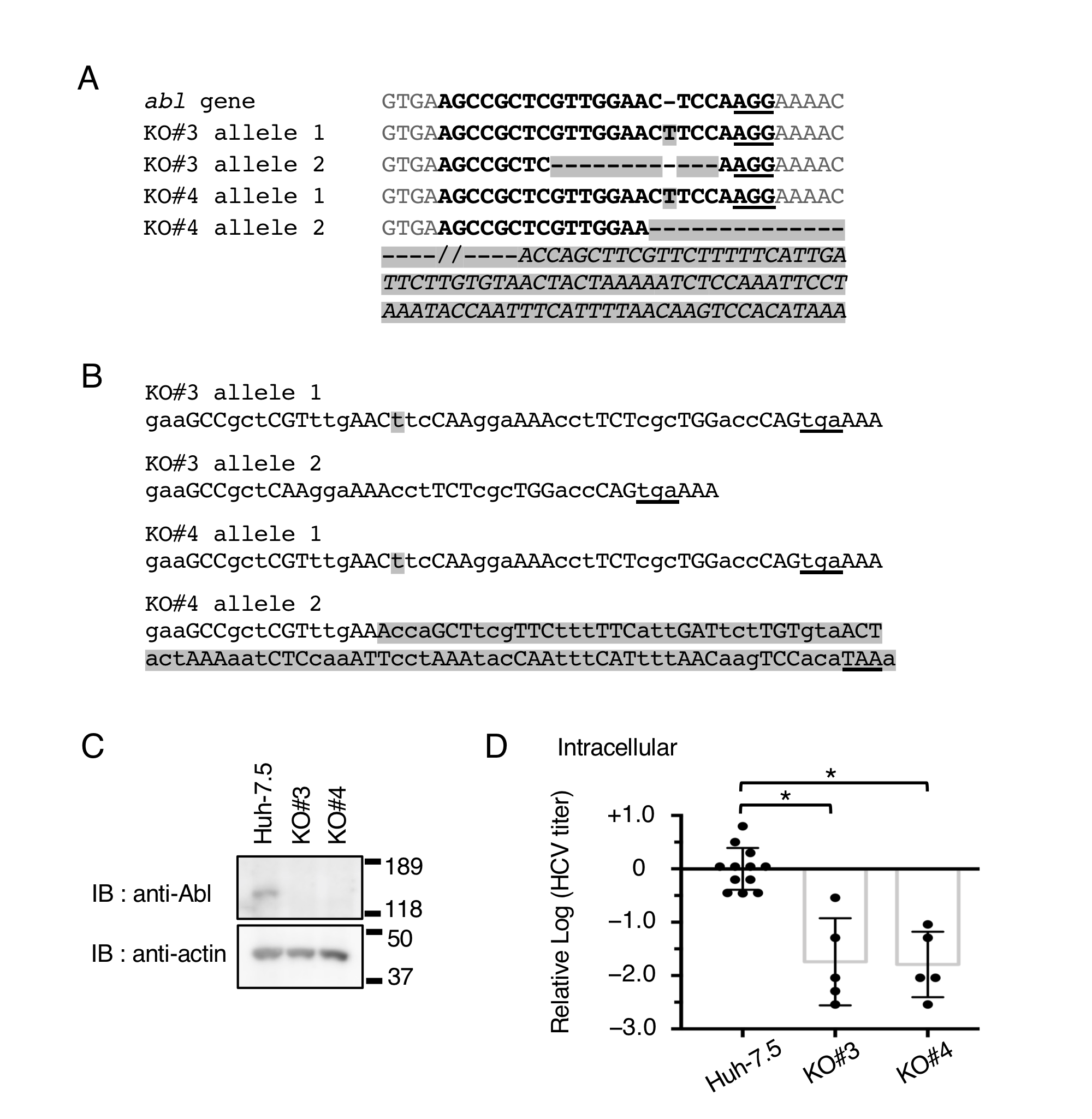


**Figure S2. Analysis of Abl^−^ cells generated using another method of transfection.**

*A*. Comparison of the nucleotide sequences of *abl* in Huh-7.5 cells and Abl^−^ cells (KO #3 and KO #4). The shaded letters are mutations caused by genome editing. The regions flanked by gray letters are the target sequence of guide RNA, which are different from those used to establish KO#1 and KO#2 shown in Fig. 1. Underlined letters indicate protospacer adjacent motif (PAM) sequences. Italic letters indicate inverted sequence of intron between exon 2 and 3 inserted in allele 2 of KO#4 cells. *B*. In-frame termination codons. The shaded letters are mutations caused by genome editing. Underlined letters indicate in-frame termination codons. *C*. Immunoblotting. The DSL of Huh-7.5 cells, KO#3, and KO#4 cells were separated via SDS-PAGE and analyzed via immunoblotting with the anti-Abl mAb and anti-actin mAb as the internal control. Molecular size markers are shown on the right. *D*. Infectious particle production. Dilution series of intracellular virus suspensions recovered from each cell line were prepared and analyzed as described in Experimental Procedures. The results are from 5 to 12 independent experiments and are presented as scatter plots with standard deviation. Asterisks indicate significant differences between paired values: *, *p* <0.05.

Abl: Abelson, Abl^−^: Abl-deficient, DSL: detergent-soluble lysate


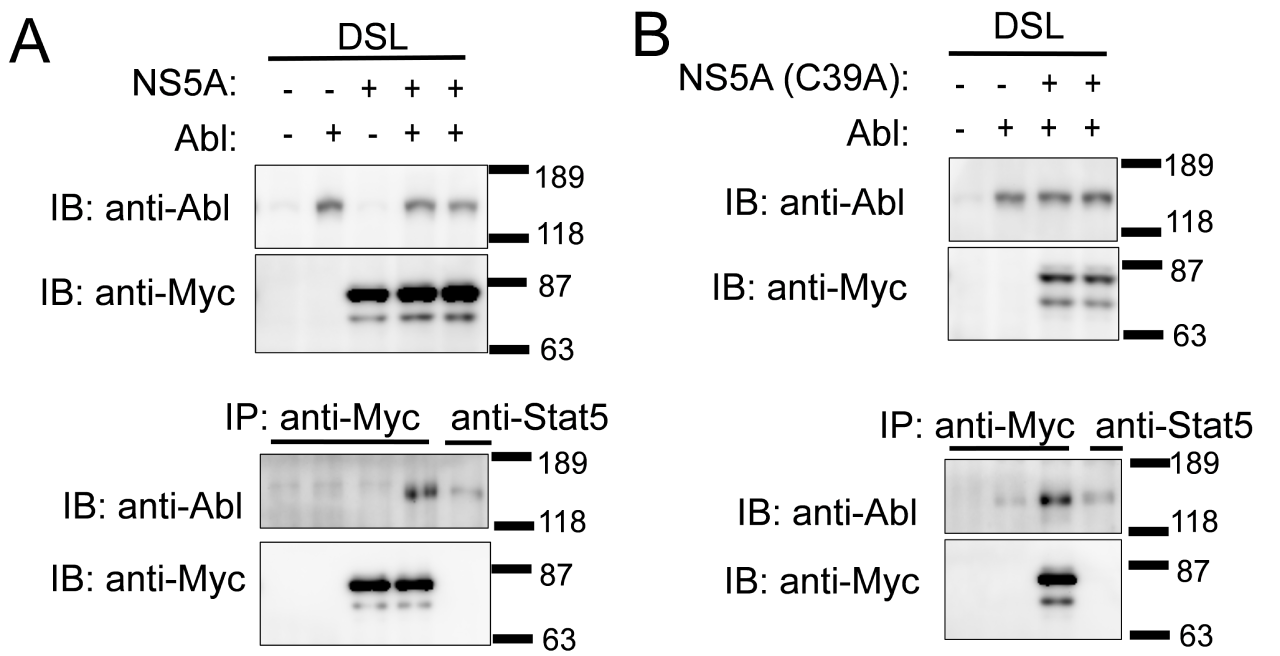


**Figure S3. Control experiments using anti-Stat5 mAb.**

HEK 293T cells were transiently transfected with a combination of pcDNA3-Abl-WT, pEF1-NS5A-WT/myc-His A (*A*), or pEF1-NS5A-C39A/myc-His A (*B*). Cells were lysed in lysis buffer lacking DOC and SDS. DSL, anti-Myc and anti-Stat5b (G-2) (Santa Cruz Biotechnology, catalog number sc-1656, lot number E279) immunoprecipitates were separated using SDS-PAGE and analyzed via immunoblotting with the indicated antibodies. Molecular size markers are shown on the right. Anti-Stat5 mAb failed to detect sufficient Abl in the immunoprecipitates.

Abl: Abelson, DOC: sodium deoxycholate, DSL: detergent-soluble lysate, NS5A: nonstructural protein 5A


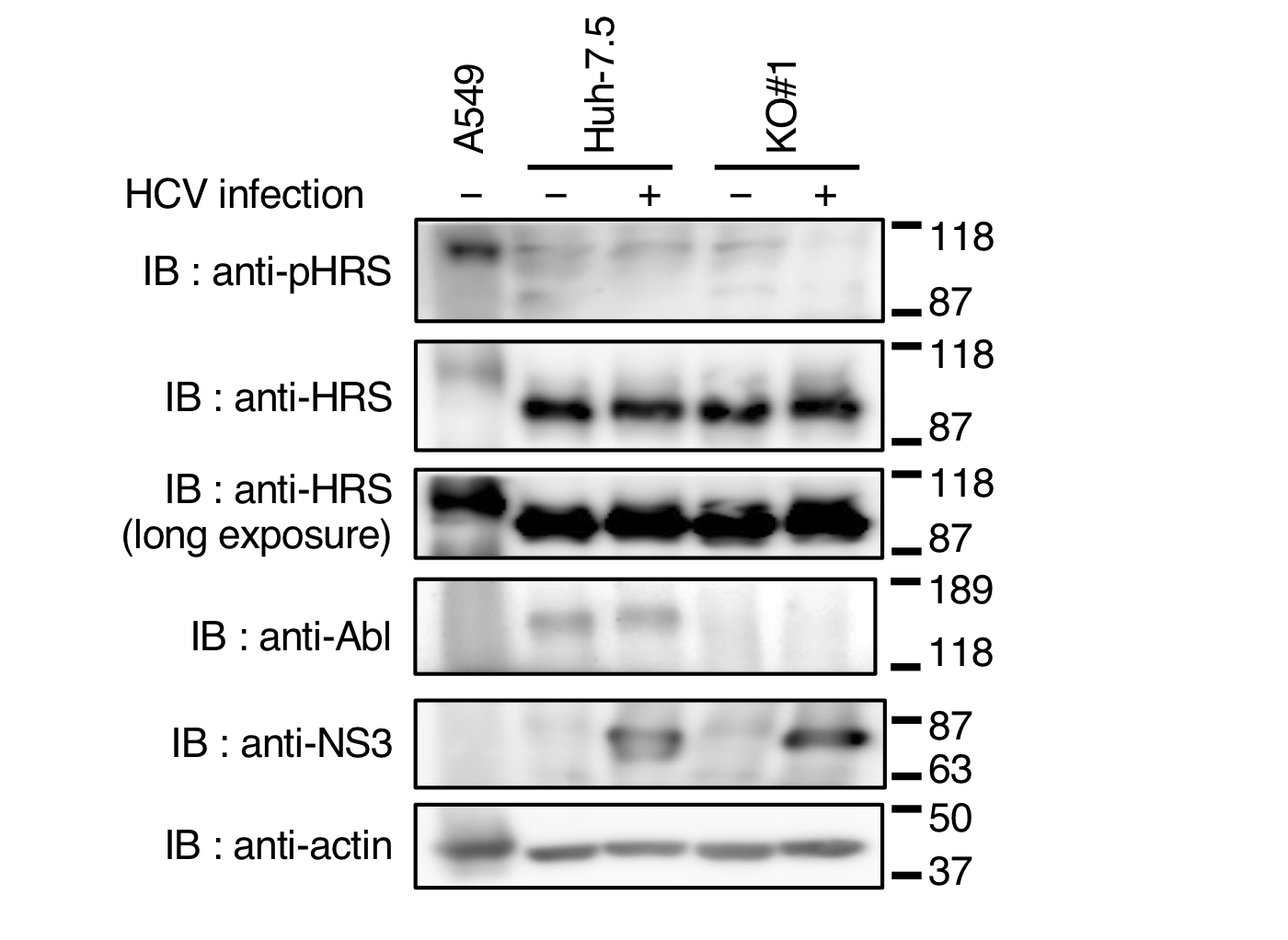


**Figure S4. Analysis of HRS phosphorylation in HCV-infected cells.**

Huh-7.5 and Abl^−^ (KO#1) cells were infected with HCVcc (J6/JFH1) at an MOI of 5. Seventy-two hours after infection, cells were lysed in lysis buffer, and DSLs were separated using SDS-PAGE and analyzed via immunoblotting with anti-phospho-HRS/HGS (Thy216) antibody (anti-pHRS, Thermo Fisher Scientific, catalogue number PA5-40200, lot number UL2908842), anti-HRS (D7T5N) mAb (Cell Signaling Technology, catalogue number 15087, lot number 1), anti-c-Abl mAb, anti-hepatitis C Virus NS3 protein polyclonal antibodies, and anti-β-actin mAb. Immunoblot with anti-HRS mAb shows additional long exposure. A549 cell lysate (Santa Cruz Biotechnology, catalogue number SC-2413, lot number H0520) was used as a positive control for immunoreaction with anti-pHRS. Molecular size markers are shown on the right. The increase in HRS phosphorylation by HCV infection was not detected in Huh-7.5 and Abl^−^ (KO#1) cells.

Abl: Abelson, Abl^−^: Abl-deficient, DSL: detergent-soluble lysate, HCV: hepatitis C virus, HCVcc: cell culture-adapted HCV


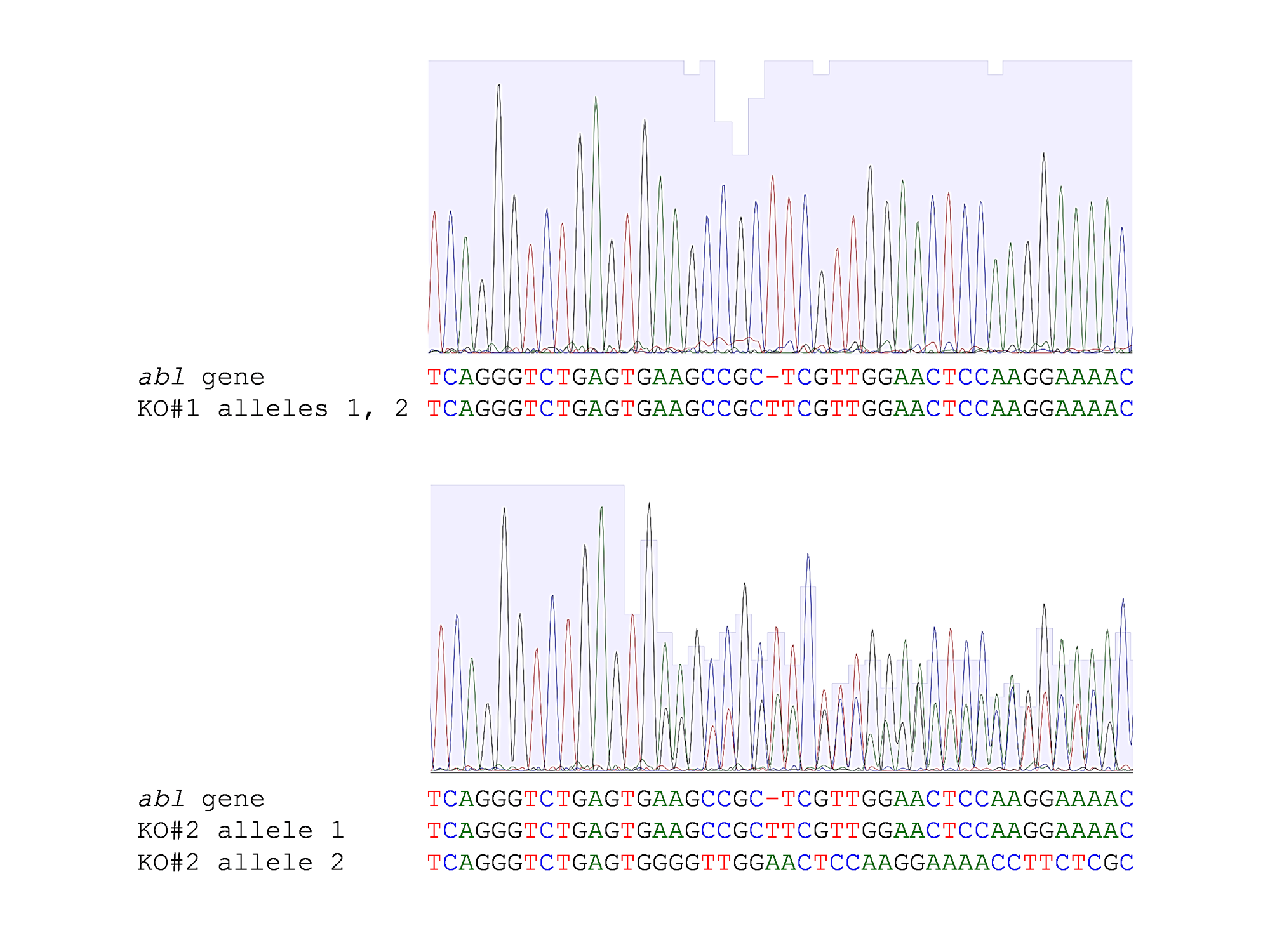


**Figure S5. Direct DNA sequence analysis of the *abl* gene in Abl^−^ cells.**

The *abl* gene in KO#1 and KO#2 cells were analyzed using direct DNA sequencing. One sequence type was identified in KO#1 cells, and two sequence types were identified in KO#2 cells. For each cell, the DNA sequence results from the subcloned alleles are merged at the bottom.

Abl: Abelson, Abl^−^: Abl-deficient


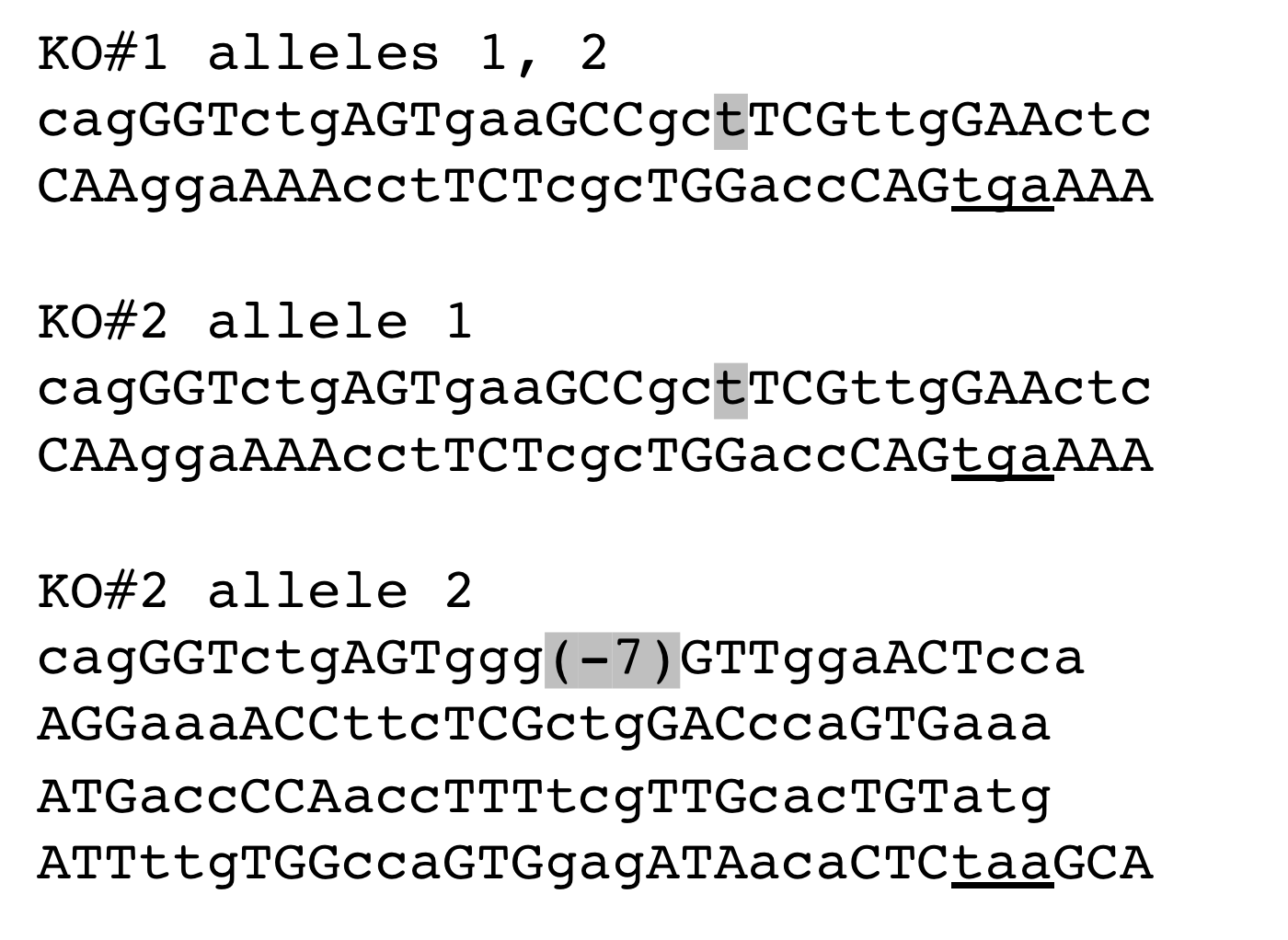


**Figure S6. In-frame termination codons generated by genome editing in the *abl* gene of Abl^−^ cells.**

Sequence analysis of the *abl* gene in KO#1 and KO#2 cells. The shaded letters are mutations caused by genome editing. Underlined letters indicate in-frame termination codons.

Abl: Abelson, Abl^−^: Abl-deficient
